# Soil pollution indices and health risk assessment of metal(loid)s in the agricultural soil of pistachio orchards

**DOI:** 10.1038/s41598-024-59450-4

**Published:** 2024-04-18

**Authors:** Mahmoud Taghavi, Khadije Bakhshi, Ahmad Zarei, Edris Hoseinzadeh, Abdolmajid Gholizadeh

**Affiliations:** 1 Department of Environmental Health Engineering, Social Determinants of Health Research Center, School of Public Health, Gonabad University of Medical Sciences, Gonabad, Iran; 2 School of Public Health, Gonabad University of Medical Sciences, Gonabad, Iran; 3 Department of Environmental Health Engineering, School of Public Health, Infectious Diseases Research Center, Gonabad University of Medical Sciences, Gonabad, Iran; 4 Department of Environmental Health Engineering, Saveh University of Medical Sciences, Saveh, Iran; 5 Department of Environmental Health Engineering, Torbat Heydariyeh University of Medical Sciences, Torbat-e Heydariyeh, Iran

**Keywords:** Human health risks, Metal(loid)s, Pistachio orchards, Pollution indices, Soil contamination, Cancer, Environmental sciences, Environmental social sciences, Diseases, Chemistry

## Abstract

Elevated levels of metal(loid)s in soil may pose potential threats to the ecosystem and can be harmful for human health. The concentrations of As, Cd, Pb, Cr and Ni were determined in agricultural soil collected from 45 pistachio orchards around Feizabad city, Khorasan Razavi province, Iran using ICP-OES. Also, soil pollution indices including contamination factor (CF), pollution load index (PLI) and geo-accumulation index (Igeo) were evaluated. In addition, non-carcinogenic and carcinogenic risk indices were estimated. The mean concentrations of metal(loid)s were in the order of Ni = 466.256 > Cr = 120.848 > Pb = 12.009 > As = 5.486 > Cd = 0.394 mg/kg. Concentrations of As, Cd and Pb in the soil samples were within their respective permissible limits set by World Health Organization (WHO). But concentrations of Cr and Ni in 84.4 and 100% of the samples, respectively exceeded the WHO allowable limits. The CF, PLI and Igeo showed that soil of some of the pistachio orchards was contaminated with some metals. The possible sources of the metals in the soil are application of pesticides, chemical fertilizers, manures as well as irrigation water. Hazard quotient (HQ) ad Hazard index (HI) values from soil of all the orchards were found to be well below the respective threshold limit (1), suggesting that there is no immediate non-cancer threat arising from the contamination at all the orchards with metal(loid)s for children and adults. The highest cancer risk values (1.13E-02 for children and 1.25E-03 for adults) were estimated for Ni in the soil. Collectively, this study provides valuable information to improve the soil in the pistachio orchards to reduce metal(loid)s contamination and minimize the associated health risks to the population in the area.

## Introduction

Soil is as a complex, living, continuously changing and dynamic component in the ecosystem and is very important for mankind survival and social development and its quality greatly affects food safety, crops product quality and eventually human health^1^. In the recent decades, the rapidly development of urbanization and industrialization has polluted soil and environment^2–4^. Degradation of soil quality by different pollutants have been increasingly recognized as a threatening environmental issue in many countries including Iran^5–7^. Among different soil pollutants, a special concern is focused on metal(loid)s. Metals are inorganic elements with densities above 5 g/cm^3^ and are classified as heavy metals. These metals are including arsenic, cadmium, chromium, cobalt, copper, lead, mercury, nickel, uranium and zinc^8^. Metal(loid)s may enter soil from natural factors (the geology/lithogenic inputs, the geographical characteristics, and local climate) or human activities. The natural levels of metal(loid)s in soil is usually low. But, human activities may change the basic characteristics of soil causing metal accumulation in the soil resulting in high levels of contamination^9^. Soils in many areas have contaminated by the accumulation of metal(loid)s (Cd, Pb, Cr, As, Hg, Cu, Ni, Zn, Al, and so on) via the emissions from human activities including the continuously expanding industrial zones, mine tailings, disposal and discharges of metal wastes, leaded gasoline and paints, land use of chemical fertilizers, organic manures, raw wastewater sludge, pesticides (insecticides, fungicides, rodenticides, nematicides, and herbicides), wastewater irrigation, coal combustion wastes and ash, release of petrochemicals, and atmospheric precipitation^10–14^. Different types of chemical fertilizers including P fertilizers, compound fertilizers, K fertilizers and N fertilizer are being used for improving the growth and yielding of crops. Amongst, P fertilizer has the highest amounts of metals such as cadmium, cobalt, copper, lead, zinc, chromium, and nickel^15,16^. Livestock manures (poultry, cattle, pig, sheep and goat farming manures) may contain elevated levels of metal(loid)s such as copper, zinc, cadmium, nickel, chromium, arsenic, lead, and mercury^17–19^. Sludge originated from domestic and industrial wastewater treatment plants has been reported contaminated with metal(loid)s including arsenic, cadmium, chromium, copper, lead, mercury, nickel, molybdenum, zinc, etc.^20,21^. The commonly reported metal(loid)s in the active components of pesticides are including copper, arsenic, lead, mercury, chromium, zinc, aluminum, lithium, barium, boron, and titanium^22^.

When metal(loid)s accumulate to high concentrations in agricultural soils, these toxic and non-degradable elements seriously affect crop health and productivity^16,23^. The toxicity of the metal(loid)s on crops vary based on the plant type, growth condition, and developmental stage, amount of toxicity of the specific elements in soil, soil physical and chemical parameters, distribution and bio-availability of metal elements in the soil environment; and the chemistry of zone of soil surrounding a plant^16^. Metal(loid)s are persistence and non-degradable in environment. Accumulation of elevated levels of these elements (e.g., lead, chromium, arsenic, zinc, cadmium, copper, mercury and nickel) in arable soils may not only result in soil contamination, but also may contribute to their high uptake by plants and crops paving their way into food chain, and thus can affect food quality and safety^24^. Since there is no good mechanism for their elimination or due to their long biological half-lives of elimination from the human or animal bodies after intake, they pose obvious health risks as a result of prolonged intake and their accumulation^25^. Although some metals such as Cu, Fe and Zn, are classified as micronutrients if found in low quantities, several metal(loid)s such as mercury, lead, arsenic, and cadmium, are non-essential for metabolic processes in the human body, and these elements are categorized as noxious^26,27^. Researches have reported that long-term exposure to As through inhalation route may cause bronchial cancer. Moreover, chronic intake of this element through ingestion may result in lung, skin and bladder cancers^28^. Exposure to high levels of chromium may cause cancer and tumor in respiratory system^29^. Ni, a possible human carcinogen, is a metal intaking mainly by humans via the respiratory route and can cause chronic bronchitis and asthma^30^. Intake of high levels of Pb may contribute to a range of harmful effects on the human body, including teratogenicity, decrement of hemoglobin synthesis, and destruction of the central and peripheral nervous systems in children, anemia, gastrointestinal disorders and urinary tract, anoxia, high blood pressure and joint/ muscle problems in pregnant women^31^. The excessive amounts of cadmium in soil has toxic effects on the beneficial microbes, damage their metabolic processes, and prevent their growth. Cadmium has a lengthy biological half in range of 10–35 years in human body^32^. Therefore, soil contamination with metals is considerably threatening people wellbeing both in developed and developing nations. Therefore, it is very important to monitor soil in order to detect polluted areas and, as far as possible, prevent progressive soil degradation. So far, there is no study regarding levels of metal(loid)s in soil of pistachio orchards in Feizabad city, Khorasan Razavi province, Iran using toxicity indices. In a previous study, metal(loid)s including zinc, mercury, cadmium, lead, copper, nickel, arsenic, and chromium were reported among the most commonly found elements in soil^33^. Therefore, the main objectives of the present study is to: (1) determine concentrations of metal(loid)s including As, Cd, Pb, Cr and Ni in the soil of pistachio orchards; (2) estimate the dietary intake of metal-polluted soil; (3) evaluate soil contamination using some soil pollution indices; and (4) assess the non-carcinogenic and carcinogenic human health risks for children and adults through various exposure pathways. The main aim of the present study was to understand the actual impact of metal(loid)s contamination on ecological and human health risks in the area and to provide more effective information for local government to manage orchards and to properly prevent soil metal(loid)s contamination.

## Materials and methods

### Study area

The study area is pistachio orchards around Feizabad city located in Khorasan Razavi province, in northeast of Iran. Iran is the second biggest producer of pistachio after the United States of America and the top exporter of this crop and supplies above 50% of the global pistachio market. In the recent years, it has become the first pistachio producing area in Khorasan Razavi (second in Iran after Kerman). Pistachio orchards in the present study cover an area of approximately 25,000 hectares. Total produced pistachio in the area is 100,000–110,000 tons annually. Some of the pistachio is marketed inside Iran and the remaining is exported to Russia, Pakistan, Uzbekistan, Kazakhstan, Afghanistan and Yemen. Pistachio trees usually grow in warm, dry climates with hot summers and cool, wet winters. Generally, pistachio trees are highly tolerant to saline soil and can grow in almost any type of soil, but well-drained light, sandy and loamy soil is preferred. In the study sites, irrigation water for pistachio orchards are extracted from deep wells (90–160 m). Each pistachio farm has its own water well. The area is located at an elevation of 1776 m above sea level. The maximum annual temperature reaches 43 °C, and the mean annual rainfall is in the range of 170–270 mm. The dominant soil texture class in the area is loamy. The main occupations of the population of Feizabad are farming and trading, more especially pistachio. There are no major industries developed within and around the study area. Owners of the orchards use chemical fertilizers and also livestock manure to enhance soil fertility. Also pesticides such as Diazinon, Ethion, Chlorpyrifos, Bordeaux, Copper Oxychloride, Dimethomorph, Thiacloprid and Fenoxycarb are usually being used for pest control during four seasons of a year. Agriculture (pistachio, saffron and wheet) and livestock (sheep, goat, poultry farm and dairy farm) are the main sources of income of the area’s active population. The Industry in the area is rarely developed.

### Sampling and analysis

**Figure 1:**
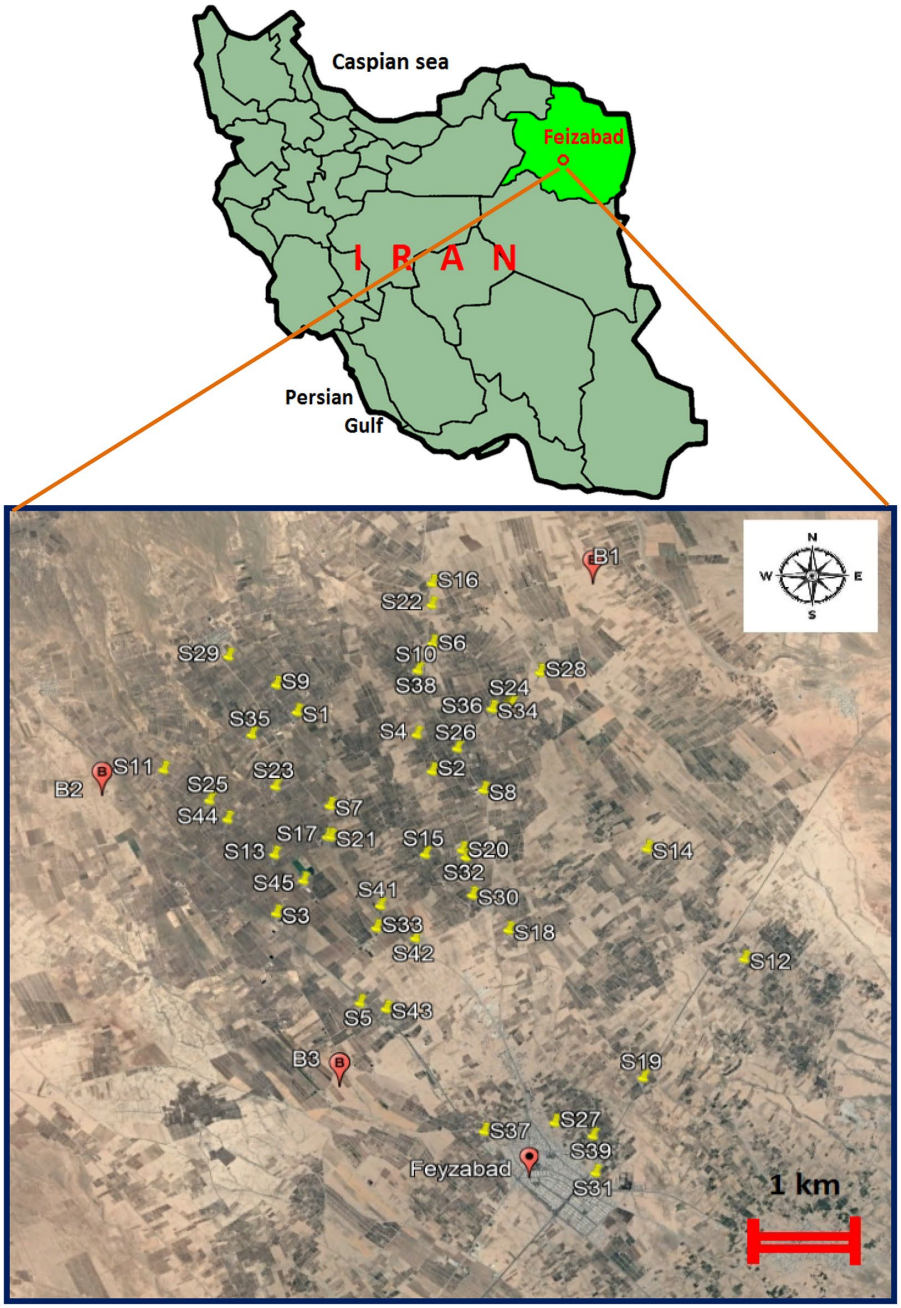
The distribution of the sampling orchards of Feizabad (*source*: https://www.google.com/search?q=feizababd+iran&client#eim=CAE).

Totally 45 orchards were selected for the purpose of the present study in October 2023. Soil sampling sites were distributed in the way to cover all the area that was under agricultural use for pistachio cultivation. The distribution of the sampling orchards of Feizabad are shown in Fig. 1. Firstly, each orchard was divided into 3 parts. Then, 3 samples from different soil layers (0–10, 10–30, and 30–50 cm) were taken from each part (totally 9 samples from each orchard). Upon soil collection, the soil samples from each orchard were mixed thoroughly to form a representative and composite soil sample (totally 45 composite samples). Three control samples were collected in the same manner at a mean distance of 100 m from orchards with no human activity (areas with no agriculture, fertilizers and poisons use and also far from urban areas and industries). The sampling sites are shown in Fig. 1, using B1–B3 codes. The soil samples were stored in clean poly vinyl chloride (PVC) bags with sample information labels. Then, they were brought to the chemistry laboratory during 24 h of sampling for further analysis. The soil samples were sun dried, dirt, stones, plant roots, and residues were eliminated and then completely ground by a porcelain pestle and mortar to pass through a two millimeters nylon screen. Then, the soil samples were analyzed for pH, electrical conductivity (EC) and metal(loid)s. Briefly, for the digestion of each soil sample, 4.5 mL 65% (m/m) HNO_3_ and 0.5 mL 30% (m/m) H_2_O_2_ was added to 1 g of the each prepared soil sample at temperature 70 °C^34^. Digested samples were diluted to 50 mL with double deionized water. The As, Cd, Pb, Cr and Ni elements were analyzed with an inductively coupled plasma optical emission spectrometer (ICP-OES 730-ES). The limits of detection (LOD) of ICP-OES were 0.006, 0.0001, 0.003, 0.0005, and 0.0003 mg/L for arsenic, cadmium, lead, chromium, and nickel, respectively. Correlation coefficient (*R*^2^) values for all the selected metal(loid)s in this study were above 0.992.

### Calculation of the SAR (sodium adsorption ratio)

The sodium adsorption ratio in the study is calculated based on the Eq. (1):

1 $${\text{SAR}} = \frac{{{\text{Na}}}}{{\sqrt {\frac{{{\text{Ca}} + {\text{Mg}}}}{2} } }}$$

where Na, Ca and Mg are sodium, calcium and magnesium in milliequivalent/liter [meq/L) or mmol/L.

### Soil pollution indices

Pollution of metal(loid)s in soil environment is a growing problem globally and currently it has reached an alarming rate in some areas. In recent decades, soil environment is highly contaminated by metal(loid)s due to the increase in the human pressure on land, industrialization, use of different types of fertilizers and other anthropogenic activities on the ecosystem. The elements accumulate and enrich the soils and sediments^35,36^. The present research aims to objectively and reasonably evaluate the status of metal(loid)s contamination (As, Cd, Pb, Cr and Ni) in the soil of pistachio orchards using three different soil contamination indices including contaminant factor, pollution load index and geo-accumulation index. These indices are important tools that have become widely accepted and used in previous studies^37–39^.

### Contamination factor

Contamination factor (CF) is a good tool showing the contamination level of soil. It is calculated using the Eq. (2) ^40^:

2 $${\text{CF}}= \frac{{{\text{C}}}_{{\text{i}}}}{{{\text{B}}}_{{\text{i}}}}$$

where ${{\text{C}}}_{{\text{i}}}$ is the level of the tested metal in the soil sample and ${{\text{B}}}_{{\text{i}}}$ is the background metal concentration in the soil, which is a baseline concentration in the soil in place/s not affected by human activities. In this study, levels of background metal(loid)s in mg/kg are: As = 10.346, Cd = 0.409, Pb = 20.08, Cr = 41.88 and Ni = 68, which is the mean level of each selected metal in three sampling sites nearest to the study area with no human activity. Classes of contamination describing CF values are given in Table 1.

**Table 1:** Classes of contamination for CF, PLI and Igeo.

Index	Value	Ecological risk	Reference/s
${\text{CF}}$	${\text{CF}}$< 1	Low contamination	^41^
	1<${\text{CF}}$ < 3	Moderate contamination	
	3<${\text{CF}}$ < 6	Considerable contamination	
	${\text{CF}}$> 6	Very high contamination	
${\text{PLI}}$	${\text{PLI}}$< 1	Represents uncontaminated soil	^42^
	1 ≤ ${\text{PLI}}$ < 2	Shows uncontaminated to moderately contaminated soil	
	2 ≤ ${\text{PLI}}$ < 3	Implies moderately to strongly contaminated soil	
	${\text{PLI}}$≥ 3	Describes strongly contaminated soil	
${{\text{I}}}_{{\text{geo}}}$	${{\text{I}}}_{{\text{geo}}}$≤ 0	Represents practically un-contaminated soil	^9,43^
	0 ≤ ${{\text{I}}}_{{\text{geo}}}$ < 1	Shows uncontaminated to moderately contaminated soil	
	1 ≤ ${{\text{I}}}_{{\text{geo}}}$ < 2	Shows moderately contaminated soil	
	2 ≤ ${{\text{I}}}_{{\text{geo}}}$ < 3	Gives moderately to heavily contaminated soil	
	3 ≤ ${{\text{I}}}_{{\text{geo}}}$ < 4	Means heavily contaminated soil	
	4 ≤ ${{\text{I}}}_{{\text{geo}}}$ < 5	Implies heavily to extremely contaminated soil	
	${{\text{I}}}_{{\text{geo}}}$≥ 5	Shows extremely contaminated soil	

### Pollution load index

All the studied orchards were estimated for the range of metal(loid)s contamination using the formula proposed by Thomilson et al. in 1980^44^. This equation evaluates overall soil toxicity. Pollution load index (PLI) is computed by the Eq. (3):

3 $${{\text{PLI}}{=({\text{CF}}}_{1}\times {{\text{CF}}}_{2 }{\times {\text{CF}}}_{3}\times \dots \times {{\text{CF}}}_{\mathrm{n }}) }^{1/n}$$

where n is the number of metal(loid)s selected for the purpose of the study (5 in the present study), and CF is the contamination factor computed using the Eq. (2). The classification of PLI is given in Table 1.

### Geo-accumulation index

Geo-accumulation index (Igeo) was suggested by Muller, a scientist from Germany, in 1969^45^. Hakanson suggested that ${{\text{I}}}_{{\text{geo}}}$ could be used to evaluate the status of metal(loid)s contamination in both land and water ecosystem. This index has been used for the evaluation of soil contamination status in many studies in different countries. ${{\text{I}}}_{{\text{geo}}}$ can be calculated using the Eq. (4):

4 $${{\text{I}}}_{{\text{geo}}}=\mathrm{Log }(\frac{{{\text{C}}}_{{\text{n}}}}{1.5{{\text{B}}}_{{\text{n}}}})$$

where ${{\text{C}}}_{{\text{n}}}$ is the concentration of the selected element in the soil sample and ${{\text{B}}}_{{\text{n}}}$ is background value of the selected element in the soil.

### Health risk assessment

In this study, the proposed method of health risk assessment of USEPA was used for the evaluation of the possible human health risks from exposure to metal(loid)s. Human health risks via exposure to contaminants can be divided in two classes: non-cancer and cancer risk. Non-cancer risk can be estimated for both carcinogen and non-carcinogen pollutants. But cancer risk is estimated only for carcinogens. For this purpose, the level of each contaminant was determined and then, health risk was estimated qualitatively and quantitatively using Eqs. (5)–(11). In this study, both non-cancer and cancer risks were assessed through three exposure pathways including ingestion, inhalation and dermal contact to soil metal(loid)s for two age groups of children (3–12 years old) and adults (18–40 years old) in the study area. Firstly, chronic daily intake (CDI) values were calculated for each element by use of Eqs. (5)–(7). Secondly, then non-cancer risk was estimated based on the hazard quotient (HQ) and hazard index (HI) as given in Eqs. (8) and (9). Finally, carcinogenic risk (CR) of each carcinogen element was calculated using Eqs. (10) and (11). The health risk assessment was estimated by use of the following equations:

5 $${{\text{CDI}}}_{{\text{ing}}}={{\text{C}}}_{{\text{soil}}}\times \frac{{\text{IngR}}\times {\text{EF}}\times {\text{ED}}}{{\text{BW}}\times {\text{AT}}}\times {10}^{-6}$$

6 $${{\text{CDI}}}_{{\text{inh}}}={{\text{C}}}_{{\text{soil}}}\times \frac{{\text{InhR}}\times {\text{EF}}\times {\text{ED}}}{{\text{PEF}}\times {\text{BW}}\times {\text{AT}}}$$

7 $${{\text{CDI}}}_{{\text{derm}}}={{\text{C}}}_{{\text{soil}}}\times \frac{{\text{SA}}\times {\text{AF}}\times {\text{ABS}}\times {\text{EF}}\times {\text{ED}}}{{\text{BW}}\times {\text{AT}}}\times {10}^{-6}$$

8 $${{\text{HQ}}}_{{\text{ing}}/{\text{inh}}/{\text{derm}}}=\frac{{{\text{CDI}}}_{{\text{ing}}/{\text{inh}}/{\text{derm}}}}{{{\text{Rfd}}}_{{\text{ing}}/{\text{inh}}/{\text{derm}}}}$$

9 $${\text{HI}}={\sum }_{{\text{k}}=1}^{3}{\text{HQ}}$$

10 $${{\text{CR}}}_{{\text{ing}}/{\text{inh}}/{\text{derm}}}={{\text{CDI}}}_{{\text{ing}}/{\text{inh}}/{\text{derm}}}.{\text{CSF}}$$

11 $$\mathrm{Total\, carcinogenic\, risk} ({\text{TCR}})={\sum }_{{\text{k}}=1}^{3}({{\text{CR}}}_{{\text{ing}}}+{{\text{CR}}}_{{\text{inh}}}+{{\text{CR}}}_{{\text{derm}}})$$

Parameters and input assumptions for exposure assessment of metal(loid)s via different pathways are given in Table 2. Also, reference doses (Rfds) and cancer risk factors (CSFs) used for health risk assessment in this research are shown in Table 3. For non-cancer risk, a HQ or HI value above 1 contributes to health risk. For carcinogens, when the CR/CRt < 1.00E-06, the risk of cancer is very low and can be neglected. Whereas, the values of CR/CRt > 1.00E-04 can cause cancer. Also when 1.00E-06 < CR/CRt < 1.00E-04, the cancer risk is low^46,47^.

**Table 2:** Parameters and input assumptions for exposure assessment of metal(loid)s via ingestion, inhalation and dermal pathways^12^.

Exposure parameters	Description	Unit	Values for each group	
			Children	Adults
${{\text{C}}}_{{\text{soil}}}$	Metal concentration in soil	mg/kg	–	–
${\text{IngR}}$	Ingestion rate	mg/day	200	100
${\text{InhR}}$	Inhalation rate	m^3^/day	7.6	20
${\text{EF}}$	Exposure frequency	Days/year	365	365
${\text{ED}}$	Exposure duration	Years	6	24
${\text{BW}}$	Body weight	Kg	15	70
${\text{AT}}$	Average timing	Days	2190	8760
${\text{SA}}$	Skin area	cm^2^	2800	5700
${\text{ABS}}$	Dermal adsorption factor	No unit	0.001	0.001
${\text{AF}}$	Adherence factor of soil	mg/cm^3^/day	0.2	0.07
${\text{PEF}}$	Particulate emission factor	m^3^/kg	1.36E+09	1.36E+09

**Table 3:** Rfds and CSFs used for health risk assessment in this research^48–50^.

Metal	Non-cancer risk			Cancer risk		
	Rfding	Rfdinh	Rfdderm	CSFing	CSFinh	CSFderm
As	3.00E-04	1.23E-04	3.01E-04	1.50E + 00	1.51E + 01	3.66E + 00
Cd	1.00E-03	1.00E-03	1.00E-05	3.80E-03	6.30E + 00	-
Pb	3.50E-03	3.52E-03	5.25E-04	8.50E-03	4.20E-02	-
Cr	3.00E-03	2.86E-05	5.00E-05	5.00E-01	4.20E + 01	2.00E + 01
Ni	2.00E-02	2.06E-02	5.40E-03	1.70E + 00	-	4.25E + 01

The risk assessment in the current work is based on the mean concentrations of metal(loid)s in the soil of pistachio orchards.

### Statistical analysis

All statistical analyses were done using excel 2016 and SPSS 2022. Pearson’s correlation coefficient was used to determine the relationships between the metal(loid)s in the samples.

## Results and discussion

### Levels of pH, EC, SAR and metal(loid)s in the soil of pistachio orchards

The pH is an important chemical property of soil as it affects solubility, bioavailability and translocation of metal(loid)s and nutrients in the soil. Therefore, the availability of the macronutrients and trace elements in soil depends on pH value of that soil^43,51^. Generally, most of microelements are better adsorbed by plants cultivated in acidic soils than in neutral or alkaline soils^51^. Soil pH in the studied orchards ranged from 7.1 to 8.3, indicating that soil of this area was neutral-alkaline (Table 4). This level of alkalinity in farming soil can reduces metal(loid)s retention and mobility in the soils^52,53^. Electrical conductivity (EC) is used for the indication of the salinity degree of soil and water samples^27^. Soil EC was in the range 4–9 dS/m. Pistachio tree is long-lived plant and is also tolerant to salt, but its growth and yielding is severely affected in high soil salinity and heavy texture soils^54^. The long-term use of water having high levels of salts may increase the soil salinity of the irrigated orchards and cause the soil quality degradation. This problem was reported in pistachio orchards in the south of Bardaskan, Khorasan Razavi province in Iran^55^. The sodium adsorption ratio (SAR) of soil was in the range 5–15. Soil of the area is loamy type. Descriptive statistics of the assayed metal(loid)s, viz. As, Cd, Pb, Cr and Ni, in all the soil samples from the pistachio orchards of Feizabad along with WHO limit for each element is presented in Table 4. Skewness values of As, Cd and Pb in the soil of pistachio orchards were calculated positively (except for Cr and Ni). The coefficients of skewness and kurtosis show the normal distribution characteristics of soil samples^56^. If amount of skewness is positive, it shows the average levels of metal(loid)s are less than their median levels. Inversely, a negative skewness value shows the average metal(loid)s levels is higher than their median value^57^. Among the selected elements, the highest amount of skewness was observed for lead (0.650). In general the mean levels of the metal(loid)s in the soil samples of pistachio orchards varied according to the following trend: Ni = 466.256 (range = 149.282–704.339) > Cr = 120.848 (range 60.659–187.691) > Pb = 12.009 (7.937–18.058) > As = 5.486 (range 4.08–6.946) > Cd = 0.394 (range 0.184–0.714) mg/kg, indicating that the soil samples had highest levels of Ni and inversely, Cd was the lowest. The concentrations of As, Cd and Pb in all of the soil samples investigated in this study were within the WHO recommended allowable limits of 30, 3 and 100 mg/kg, respectively. But concentrations of Cr and Ni in 84.4 and 100% of the samples, respectively exceeded the WHO recommended allowable limits (100 for Cr and 50 for Ni). Therefore, this soil is not suitable for gardening and agriculture because Cr and Ni concentrations exceeded the limits set by WHO. So it is concluded that soil is not safe from the toxic effects of Cr and Ni. High amounts of Ni and Cr, above the Iranian Environmental Quality Standard for agricultural soils, also have been reported in a previous study in agricultural soils of Kermanshah province, Iran^58^.

**Table 4:** Statistical parameters of the metal(loid)s in mg/kg (n = 45) along with soil characteristics in soil of pistachio orchards in Feizabad.

Item	As	Cd	Pb	Cr	Ni	pH	EC (ds/m)	SAR
Minimum	4.08	0.184	7.937	60.659	149.282	7.1	4.0	5.0
Mean	5.486	0.394	12.009	120.848	466.256	7.7	6.7	11.9
Maximum	6.946	0.714	18.058	187.691	704.339	8.3	9.0	15.0
Standard deviation	0.708	0.177	2.496	22.547	121.249	0.4	1.5	2.8
Skewness	0.013	0.310	0.650	− 0.304	− 0.185	0.1	− 0.2	− 0.5
Kurtosis	− 0.906	− 1.497	− 0.294	2.049	− 0.129	− 1.3	− 1.2	− 0.9
WHO guidelines	30	3	100	100	50			
Local background values	10.346	0.409	20.08	41.88	68			
% above the FAO/WHO	0	0	0	84.4	100			

Nickel is a key trace element for human and animal health, especially for manufacture of red blood cells but excessive levels can be somewhat dangerous^59^. Ni can be adsorbed easily and rapidly by plants and thus may accumulate in crops^60^. The decreasing trend of the elements (Ni > Pb > Cd) was also reported from soil of different farms in Riyadh district in Saudi Arabia^26^. Therefore, care should be taken to these elements due to their detrimental and toxic effects on human health. Our current results are higher than those obtained in Lahore, Pakistan where the mean concentrations of nickel and lead in the agricultural soil were 28.3 and 15.5 mg/kg, respectively^61^. The mean concentration of Cr in the local background study (41.88 mg/kg) was higher than a study in Rampal of Bangladesh (27.6 mg/kg)^59^. Chromium can seriously damage the lungs and kidneys in human body^62^. Inappropriate use and application of excessive levels of chemical fertilizers and pesticides in addition to increasing trend of industrial and human activities especially mining are reported as the major sources of soil contamination with a variety of toxic metals such as As, Cd, Cu, Ni, Pb and Zn^25,41,63,64^. For example, in the past, approximately 10% of the chemicals used as insecticides and fungicides in United Kingdom were containing copper, mercury, manganese, lead, or zinc. As containing matters were also used considerably to kill cattle ticks and to prevent pests in banana trees^65^. The long-term application of bio-solids such as livestock manures, composts, and municipal sewage treatment plant sludge to soil contributes to accumulation of metal(loid)s (cadmium, chromium, copper, lead, mercury, nickel, selenium, molybdenum, zinc, thallium, antimony, etc.) in soils^66^. In previous studies, the extensive use of the metal containing pesticides and fungicides (copper, zinc, lead, and arsenic) for the control of pests in crops such as apple, citrus, grape, cherry, and peach has increased accumulation of these elements in the farming soils^67,68^. Furthermore, in a study the levels of Ni and Cd were slightly high in agricultural soils associated with the commonly used pesticides (imidacloprid, acetamiprid, and emamectin) in cotton fields^69^. These toxic metals can diminish soil fertility and enter the food chain which contribute to accumulation of these elements in crops and foodstuffs and eventually can threaten human health. In another study, lead levels were high in soils (43–83 mg/kg) and also in crops (18–36 mg/kg) in Region of Kandahar in Afghanistan which was attributed to the type of minerals and groundwater quality of the area^70^. The intake of excess levels of certain amounts of the elements can cause abnormalities and health problems in human body^36,71^. Moreover, the accumulation of metal(loid)s in topsoil can migrate vertically and aggravate groundwater contamination^72^. In Iran, pesticides such as Diazinon, Ethion, Chlorpyrifos, Bordeaux, Copper Oxychloride, Dimethomorph, Thiacloprid and Fenoxycarb are usually being used for pest control during four seasons of a year in pistachio orchards which may increase the levels of metals in the soil.

In a study in Iran, mean concentrations of Cd, Ni, Cr and Pb were 2.7, 306.2, 217.5, 18.8 mg/kg, respectively in agricultural soils of southern Sabzevar in northeastern Iran^73^. Choobkar and Parsa reported mean As, Pb and Cd levels of 0.04, 8.26, 0.15 mg/kg in the agriculture soil of Zahab plain in Kermanshah, Iran^74^. In a study in agricultural soils of National Capital Region, Delhi, the mean levels of elements were reported in the decreasing order of Ni (35.34) > Cr (33.68) > Pb (18.45) > Cd (0.92)^75^. This order is similar to the result of the present study. Mean metal levels in agricultural soil of rice grain in the Dumuria Upazila under Khulna district in Bangladesh were reported as: Ni = 61.73–94.52, As = 7.53–19.63, and Pb = 15.17–29.19 mg/kg^76^. The mean concentrations of cadmium, chromium, copper, lead and zinc in an investigation in agricultural soil of northeast area of Tadla plain in Morocco were 0.92, 32.72, 138.10, 31.72, and 162.11 mg/kg, respectively, and decreased in the order of zinc > copper > chromium > lead > cadmium^53^. Mean levels of Pb and Cd in soil at agricultural areas in Kota Bharu and Bachok Districts of Kelantan of Malaysia were 26.07 and 0.66 mg/kg, respectively, which were higher than those in this study^77^. Eriksson et al. reported As, Cd, Pb, Cr and Ni, 0.25, 0.17,18, 0.22 and 13 mg/kg in agricultural soils in Sweden^78^. Ogundele et al. reported cadmium, zinc, copper, chromium, lead and nickel in soil samples as: 0.066, 9.50, 4.83, 55.63, 33.667 and 4.33 mg/kg, respectively along heavy traffic roads in North Central Nigeria. According to their study, soil and plant along road sides were contaminated with high levels of metal(loid)s^60^. In another study in Nigeria, the concentrations of Cd were in the range of 0.07–9.80, Co = 0.05–38.1, Cu = 0.33–16.9, Ni = 3.81–93.1, Pb = 4.45–47.7 and Zn = 5.02–81.4 mg/kg in agricultural soils^79^. The varying levels of metals in the soil of the present study compared to those from literature is probably due to the use of different types of pesticides and frequency use, geology of the area, higher irrigation water salinity, use of different amounts of manure and chemical fertilizers, method of metals analysis and also season of the sampling as well as plant type. Since there is no industrial activity whitin or around the study area, therefore, high levels of Cr and Ni may be probably from the area soil itself, use of different agricultural applications such as addition of manures, fertilizers and pesticides as well as irrigation water. This result is similar to the result of a previous study^10^.

### Evaluation of metal(loid)s pollution using pollution indices

#### Contamination factor

**Figure 2:**
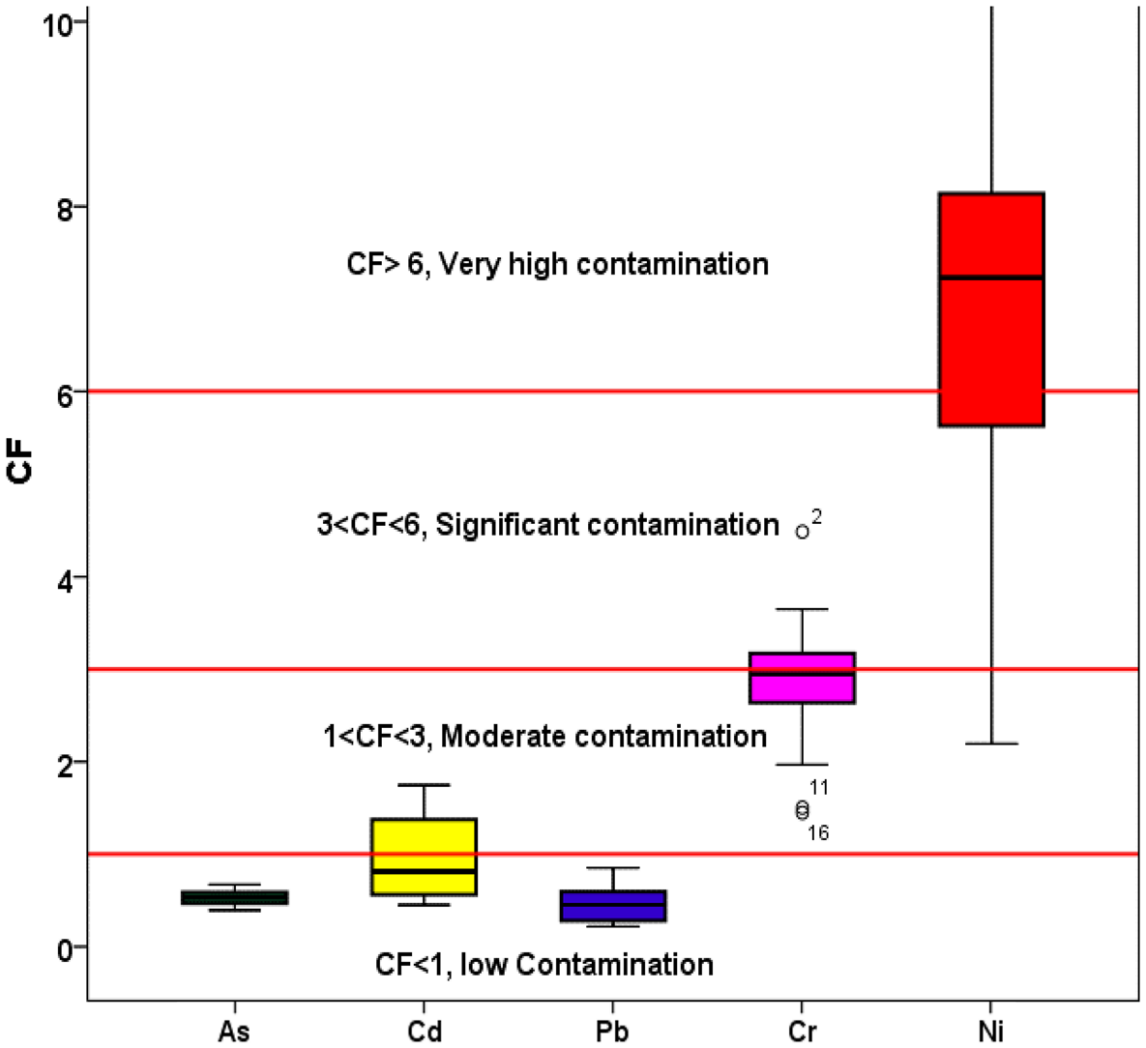
The box and whisker-plot of contamination factor (CF) values for five metal(loid)s in the soil of pistachio orchards.

Figure 2 shows the contamination factor (CF) of As, Cd, Pb, Cr and Ni in the soil of Feizabad’s pistachio orchards. The CF of the metal(loid)s in this study ranked in the order of Ni = 6.856 (2.195–10.357) > Cr = 2.885 (1.448–4.481) > Cd = 0.963 (0.449–1.745) > As = 0.530 (0.394–0.671) > Pb = 0.451 (0.218–0.851). The values of CF index for As and Pb were less than 1 in all of the pistachio orchards, which indicate that there is low overall contamination status in the soil due to the trace elements enrichment. The CF showed that Cd in the soils of 46.6% and 53.4% of orchards were in moderate contamination and low contamination status, respectively. The CF values for Cr in the soil of 62.2 and 37.8% of the orchards were in considerable contamination and moderate contamination level, respectively. For Ni, 60, 37.8 and 2.2% of orchards were in very high contamination, considerable contamination and moderate contamination status, respectively.

In agricultural soil of Morocco, the mean CF values of cadmium, chromium, copper, lead, and zinc were 1.08, 1.30, 4.40, 0.97, and 3.70, respectively. Cadmium and chromium exhibited CF values above 1 that showed moderate contamination of the investigated soils. Lead showed CF less than 1, showing low contamination of the investigated soils. However, the mean values of CF for copper and zinc were in the range 3–6, thus showing considerable contamination that would be related to the application of agricultural fertilizers in the soil^53^. In a study in agricultural soil of Saudi Arabia, CF values of Ni, Cr and Cd were above 1, indicating overall moderate contamination, while for Pb, they were < 1, indicating low contamination^80^. In a study in Delhi, sewage irrigated agricultural soils were reported to be highly contaminated with cadmium, nickel, and zinc. Moreover, soil samples near residential areas were also moderately contaminated with zinc due to the use of zinc fertilizers in order to increase crop yield^75^.

#### Pollution load index

**Figure 3:**
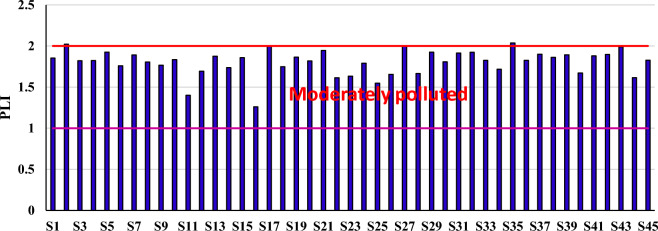
The pollution load index (PLI) in the soil of pistachio orchards.

The PLI value provides the status of metal(loid)s contamination in the soil. The pollution load index (PLI) in pistachio orchards is depicted in Fig. 3. The PLI values were in the range between 1.260 and 2.038 (mean 1.80). The PLI values of As, Cd, Pb, Cr and Ni in all of the samples, except two samples with moderately to strongly contamination, were in no contamination to moderately contamination class (1 ≤ ${\text{PLI}}\hspace{0.17em}$< 2). This result shows that metal(loid)s contamination should be taken into account during development strategies to prevent the agricultural soils of pistachio orchards from long-term pollution issues.

In agricultural soil of Morocco, the mean PLI values were reported in the range of 0.25–3.76 (mean 1.25). Totally, 36.66% of the analyzed soil samples showed PLI less than 1, arguing that the soil samples were in uncontaminated status, while 63.33% of samples showed PLI above 1, meaning its contamination with metals^53^. In a study in agricultural soil of Saudi Arabia, 4.2% of samples showed high levels (PLI> 1). Lower values of PLI imply no considerable input from anthropogenic sources^80^.

#### Geo-accumulation index

**Figure 4:**
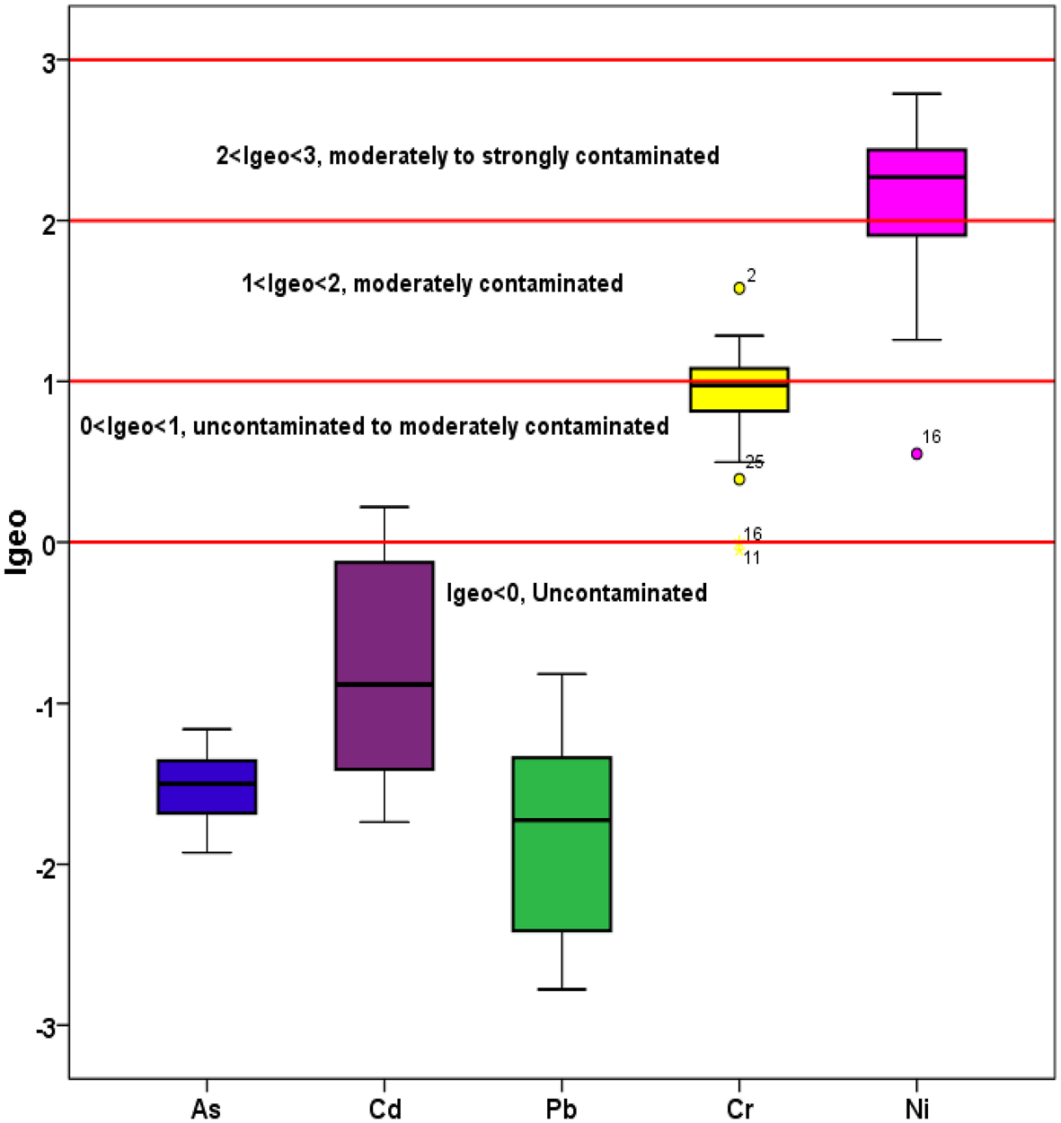
Box and whisker-plot of geo-accumulation index (Igeo) of metal(loid)s in the soil of pistachio orchards.

Generally, geo-accumulation index measures the metal(loid)s contamination degree of soil. Box and whisker-plot of geo-accumulation index (Igeo) of metal(loid)s in the pistachio orchards is shown in Fig. 4. In studied soil of the orchards, the order of Igeo for metal(loid)s in the soil was Ni = 2.136 (0.549–2.787) > As = 1.512(1.927–1.159) > Cr = 0.916 (− 0.050–1.579), Cd = − 0.787 (− 1.737–0.218), Pb = − 1.846 [(− 2.778–(− 0.816)]. The geo-accumulation index values corresponding to As and Pb for all the collected samples were negative, indicating that the pistachio orchards in the present study are practically uncontaminated due to the accumulation of these trace elements. In the case of Ni, 62.3% out of the total 45 samples showed moderately to heavily contamination (2 ≤ ${{\text{I}}}_{{\text{geo}}}$  < 3), 35.5% counted for moderately contaminated (1 ≤ ${{\text{I}}}_{{\text{geo}}}$ < 2), whereas the rest of 2.2% samples contributed no contamination to moderately contamination (0 ≤ ${{\text{I}}}_{{\text{geo}}}$ < 1). The Igeo values of Cd in 13.3 and 86.6% of the samples indicated uncontaminated to moderately contaminated and practically uncontaminated status, respectively. The Igeo values for Cr contributed about 37.7% to moderately contamination, 60% to no contamination to moderately contamination while the rest of the samples (2.2%) contributed practically no contamination. Previous studies have attributed cadmium, copper, and zinc in agricultural soil to the application of livestock manure, fertilizers, and pesticides^81–85^.

In a study in Kermanshah province, Iran, trace elements pollution in agricultural soils was assessed. The Igeo of the trace elements were in the order of Ni > Cu > Cr > Mn > Zn > Fe. The high Igeo for Ni and Cu in agricultural soils indicated that there was a considerable Ni and Cu contamination in the soils of this province^58^. In agricultural soil of Morocco, the mean Igeo levels were − 0.79, − 1.06, 0.62, − 1.97, and 0.90 for cadmium, chromium, copper, lead, and zinc, respectively. The mean Igeo values of cadmium, chromium and lead, were less than 0 (Igeo < 0), meaning that the studied soils were uncontaminated by these metals. But in the case of Cu and Zn, the soil samples were uncontaminated to moderately contaminated (0 ≤ Igeo < 1)^53^. In a study in agricultural soil of Saudi Arabia, calculated Igeo values for elements including Ni and Cr indicated that above 89% of the soil samples were in the uncontaminated status. While Igeo for Pb and Cd indicated that all of the analyzed samples were in the unpolluted status^80^. The variation of CF, PLI and Igeo for metal(loid)s observed in soil in this study could have occurred both as a result of natural processes and different man-made sources. The possible causes of high levels of these metal(loid)s, but not limited to, especially Cr and Ni could be from soil itself, application of manures, chemical fertilizers, pesticides and increase of soil salinity from irrigation water. Therefore, it is important to make farmers and government aware of this issue for enforcing proper actions in order to stop further soil degradation in pistachio orchards.

### Correlation matrix of metal(loid)s along with soil characteristics in the soil of pistachio orchards

Pearson’s correlation coefficient values for the studied metal(loid)s along with soil characteristics in the soil of pistachio orchards are presented in Table 5. As given in the table, strong correlations were found between Pb and Cd (*R*^2^ = 0.700), As and Pb (*R*^2^ = 0.619), Ni and As (*R*^2^ = − 0.618). Correlations established between the following pairs were moderate: Cd and As (*R*^2^ = 0.489), Ni and Cd (*R*^2^ = − 0.462), Ni and Pb (*R*^2^ = − 0.599), Ni and Cr (*R*^2^ = 0.561). Negative sign is an indication that two variables move in the opposite direction. Strong correlation was observed between pH and SAR, between SAR and EC, and also between pH and EC.

**Table 5:** Pearson’s correlation coefficient values of metal(loid)s along with soil characteristics in the soil of pistachio orchards.

	As	Cd	Pb	Cr	Ni	pH	EC	SAR
As	1							
Cd	0.489**	1						
Pb	0.619**	0.700**	1					
Cr	− 0.357*	0.130	− 0.236	1				
Ni	− 0.618**	− 0.462**	− 0.599**	0.561**	1			
pH	0.216	0.128	0.153	0.055	0.032	1		
EC	− 0.140	− 0.107	− 0.136	− 0.065	− 0.083	− 0.891**	1	
SAR	− 0.133	− 0.111	− 0.146	− 0.071	− 0.122	− 0.884**	0.975**	1

**Correlation is significant at the 0.01 level (2-tailed).

*Correlation is significant at the 0.05 level (2-tailed).

### Health risk assessment

#### Non-carcinogenic risk levels

The second step was to estimate the non-carcinogenic risk associated with ingestion, inhalation and dermal contact of five metal(loid)s for children and adults contained in soil samples. The mean levels of each element presented in Table 4 have been substituted into formulas (5)–(9).

HQ and HI values associated with metal(loid)s exposure for children and adults from soil is given in Table 6. Greater chances of non-cancer risk (HI) in children were observed by the metal(loid)s in the decreasing order as follows; Cr > Ni > As > Pb > Cd, while in adults, the same risk pattern was also found for the metal(loid)s. HQ or HI values from soil of all the orchards were found to be well below the respective threshold limit (1), suggesting that there is no immediate non-cancer threat arising from the contamination at all the orchards with metal(loid)s for the studied population.

**Table 6:** HQ and HI values for children and adults from metal(loid)s exposure in soil.

Risk	Pathway	As	Cd	Pb	Cr	Ni
HQ children	Ingestion	2.44E-01	5.26E-03	4.57E-02	5.37E-01	3.11E-01
	Inhalation	1.66E-05	1.47E-07	1.27E-06	1.57E-03	8.43E-06
	Dermal contact	6.80E-04	1.47E-03	8.54E-04	9.02E-02	3.22E-03
HI children		2.45E-01	6.73E-03	4.66E-02	6.29E-01	3.14E-01
HQ adults	Ingestion	2.61E-02	5.63E-04	4.90E-03	5.75E-02	3.33E-02
	Inhalation	9.37E-06	8.28E-08	7.17E-07	8.88E-04	4.75E-06
	Dermal contact	2.44E-01	2.25E-04	1.30E-04	1.38E-02	4.92E-04
HI adults		2.62E-02	7.88E-04	5.03E-03	7.22E-02	3.38E-02

#### Carcer risk

The transfer of metals from soil to human body is a matter of the concern for many scientists in different regions of the world. In this study, the cancer risk of elements including As, Cd, Pb, Cr and Ni was evaluated. The cancer risk of metal(loid)s from soil exposure is summarized in Table 7. CR total (sum of ingestion, inhalation and dermal contact pathways for each element) associated with the exposure of children to the studied elements in the soil samples of pistachio orchards were in the order of: Ni (1.13E-02) > Cr (8.98E-04) > As (1.10E-04) > Pb (1.36E-06) > Cd (2.09E-08). For adults, the order of CR total values were Ni (1.25E-03) > Cr (1.01E-04) > As (1.19E-05) > Pb (1.46E-07) > Cd (2.66E-09). Therefore, the highest cancer risk was estimated for Ni in the soil. Among the selected metal(loid)s, it was exhibited that Ni pose carcinogenic risk (CRt > 1.00E-04) for children, through oral ingestion, dermal contact, and particulate inhalation. Cr and As in the soil of the orchards showed low cancer risk (1.00E-06 < CRt < 1.00E-04). The lowest CRt values were observed for Pb and Cd in the range of negligible cancer risk (CRt < 1.00E-06).

**Table 7:** The cancer risk of metal(loid)s from soil exposure.

Metal	Children				Adults			
	CRing	CRinh	CRderm	CR total	CRing	CRinh	CRderm	CR total
As	1.10E-04	3.09E-08	7.50E-07	1.10E-04	1.18E-05	1.74E-08	1.14E-07	1.19E-05
Cd	2.00E-08	9.25E-10	-	2.09E-08	2.14E-09	5.22E-10	0.00E + 00	2.66E-09
Pb	1.36E-06	1.88E-10	-	1.36E-06	1.46E-07	1.06E-10	0.00E + 00	1.46E-07
Cr	8.06E-04	1.89E-06	9.02E-05	8.98E-04	8.63E-05	1.07E-06	1.38E-05	1.01E-04
Ni	1.06E-02	0.00E + 00	7.40E-04	1.13E-02	1.13E-03	-	1.13E-04	1.25E-03

**Figure 5:**
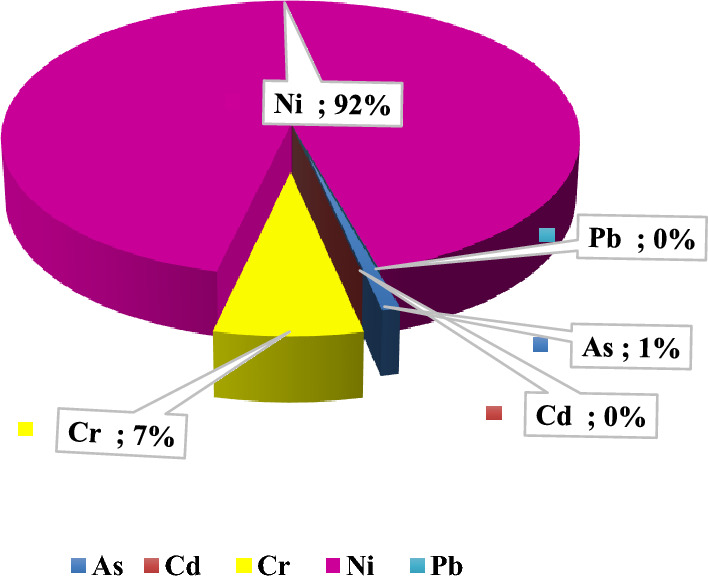
Percent contribution of each element in CRt in soil samples for all the studied orchards.

The carcinogenic health risk indicated that cancer risk of Ni is especially high if children and adults continuously be exposed to the soil from the orchards over time. It is therefore important to remediate health risks caused by metal(loid)s accumulation in pistachio orchards. Percent contribution of each metal in CRt in soil samples for all the studied orchards are shown in Fig. 5. As seen, the highest contribution is for Ni (92%).

## Conclusions

Nowadays, the pollution of agricultural soil by metal(loid)s has become a serious environmental issue globally due to potential threats to crops and eventually human health. In this study, it is found that metal(loid)s contamination in the soil of pistachio orchards was highest for Ni, followed by Cr > Pb > As > Cd, which may increase the concentrations of the metal(loid)s in the produced pistachio crop. The levels of As, Cd and Pb in all of the soil samples in the present study were within the WHO recommended allowable limits. But levels of Cr and Ni in 84.4 and 100% of the soil samples, respectively were above the WHO recommended allowable limits. The CF values of the metal(loid)s in the current study ranked in the order of: Ni = 6.856 (2.195–10.357) > Cr = 2.885 (1.448–4.481) > Cd = 0.963 (0.449–1.745) > As = 0.530 (0.394–0.671) > Pb = 0.451 (0.218–0.851). The PLI values of As, Cd, Pb, Cr and Ni in all of the samples, except two samples with moderately to strongly contamination, were in no contamination to moderately contamination class (1 ≤ ${\text{PLI}}\hspace{0.17em}$< 2). Igeo index showed soil contamination in some orchards. Values of HQ and HI from soil of all the orchards were found to be well below the respective threshold limit (1), showing that there is no immediate non-cancer threat arising from the contamination at all the orchards with metal(loid)s for the studied age groups. Among the studied metal(loid)s, it is found that Ni pose carcinogenic risk (CRt > 1.00E-04) to children, through oral ingestion, dermal contact, and particulate inhalation. The results presented in the paper can enhance our knowledge and create a benchmark to the relevant authorities regarding metal(loid)s contamination status, potential human health risks, and potential controlling factors for metal(loid)s accumulation in the soil of pistachio orchards in the studied area. It also helps in providing more comprehensive and up-to-date results for contributing to better soil management, soil remediation, and soil deterioration control. Hence, in order to safeguard food safety, a further study of metal(loid)s in pistachio crop grown in the soil of the area is highly recommended.

## Data Availability

All of the data analyzed and used during the current study will be available from the corresponding author on reasonable request.

## References

[1] Sezgin N, Kinda S, Temelli UE, Sezgin N (2023). Pollution indices assessment of metal concentrations in Karabuk soil samples. Int. J. Agric. Environ. Food Sci..

[2] Mishra RK, Mohammad N, Roychoudhury N (2016). Soil pollution: Causes, effects and control. Van Sangyan..

[3] Khan S, Naushad M, Lima EC, Zhang S, Shaheen SM, Rinklebe J (2021). Global soil pollution by toxic elements: Current status and future perspectives on the risk assessment and remediation strategies—A review. J. Hazard. Mater..

[4] Sajjadi SA, Mohammadi A, Khosravi R, Zarei A (2022). Distribution, exposure, and human health risk analysis of heavy metals in drinking groundwater of Ghayen County, Iran. Geocarto Int..

[5] Chen S-B, Meng W, Li S-S, Zhao Z-Q, Wen-di E (2018). Overview on current criteria for heavy metals and its hint for the revision of soil environmental quality standards in China. J. Integr. Agri..

[6] Faraji M, Alizadeh I, Conti GO, Mohammadi A (2023). Investigation of health and ecological risk attributed to the soil heavy metals in Iran: Systematic review and meta-analysis. Sci Total Environ..

[7] Kowalska JB, Mazurek R, Gąsiorek M, Zaleski T (2018). Pollution indices as useful tools for the comprehensive evaluation of the degree of soil contamination—A review. Environ. Geochem. Health..

[8] El-Araby EH, Salman KA, Mubarak F (2021). Human risk due to radon and heavy metals in soil. Iran. J. Public Health..

[9] Hoque MM, Islam A, Islam ARMT, Pal SC, Mahammad S, Alam E (2023). Assessment of soil heavy metal pollution and associated ecological risk of agriculture dominated mid-channel bars in a subtropical river basin. Sci. Rep..

[10] Wuana RA, Okieimen FE (2011). Heavy metals in contaminated soils: a review of sources, chemistry, risks and best available strategies for remediation. Int. Sch. Res. Not..

[11] Mahammad S, Islam A, Shit PK (2023). Geospatial assessment of groundwater quality using entropy-based irrigation water quality index and heavy metal pollution indices. Environ. Sci. Pollut. Res..

[12] Karimi A, Naghizadeh A, Biglari H, Peirovi R, Ghasemi A, Zarei A (2020). Assessment of human health risks and pollution index for heavy metals in farmlands irrigated by effluents of stabilization ponds. Environ. Sci. Pollut. Res..

[13] Huang Y, Wang L, Wang W, Li T, He Z, Yang X (2019). Current status of agricultural soil pollution by heavy metals in China: A meta-analysis. Sci. Total Environ..

[14] Vácha R. Heavy metal pollution and its effects on agriculture. MDPI 2021:1719.

[15] Luo L, Ma Y, Zhang S, Wei D, Zhu Y-G (2009). An inventory of trace element inputs to agricultural soils in China. J. Environ. Manag..

[16] Rashid A, Schutte BJ, Ulery A, Deyholos MK, Sanogo S, Lehnhoff EA (2023). Heavy metal contamination in agricultural soil: Environmental pollutants affecting crop health. Agronomy.

[17] Zhuang Z, Mu H-Y, Fu P-N, Wan Y-N, Yu Y, Wang Q (2020). Accumulation of potentially toxic elements in agricultural soil and scenario analysis of cadmium inputs by fertilization: A case study in Quzhou county. J. Environ. Manag..

[18] Kumar, V., Singh, J. & Kumar, P. Heavy metals accumulation in crop plants: Sources, response mechanisms, stress tolerance and their effects. In: Contaminants in agriculture and environment: health risks and remediation. **1**, 38 (2019).

[19] Priyanka P, Kumar D, Yadav A, Yadav K (2020). Nanobiotechnology and its application in agriculture and food production. Nanotechnology for Food, Agriculture, and Environment.

[20] Sonon, L. S. & Gaskin, J. W. Metal concentration standards for land application of biosolids and other by-products in Georgia. (2009).

[21] Zarcinas BA, Ishak CF, McLaughlin MJ, Cozens G (2004). Heavy metals in soils and crops in Southeast Asia: 1 Peninsular Malaysia. Environ. Geochem. Health..

[22] Lewis KA, Tzilivakis J, Warner DJ, Green A (2016). An international database for pesticide risk assessments and management. Hum. Ecol. Risk Assess..

[23] Peirovi-Minaee R, Alami A, Moghaddam A, Zarei A (2023). Determination of concentration of metals in grapes grown in Gonabad Vineyards and assessment of associated health risks. Biol. Trace Elem. Res..

[24] Lu Y, Yin W, Huang L, Zhang G, Zhao Y (2011). Assessment of bioaccessibility and exposure risk of arsenic and lead in urban soils of Guangzhou City, China. Environ. Geochem. Health..

[25] Weissmannová HD, Pavlovský J (2017). Indices of soil contamination by heavy metals–methodology of calculation for pollution assessment (minireview). Environ. Monit. Assess..

[26] Alturiqi AS, Albedair LA, Ali MH (2020). Health risk assessment of heavy metals in irrigation water, soil and vegetables from different farms in Riyadh district, Saudi Arabia. J. Elementol..

[27] Abdulhamid Z, Agbaji E, Gimba C, Agbaji A (2015). Physicochemical parameters and heavy metals content of soil samples from farms in Minna. Int. Lett. Chem. Phys. Astron..

[28] Papillomaviruses H (2011). IARC Monographs on the Evaluation of Carcinogenic Risks to Humans.

[29] ur rehman I, Ishaq M, Ali L, Khan S, Ahmad I, Din IU (2018). Enrichment, spatial distribution of potential ecological and human health risk assessment via toxic metals in soil and surface water ingestion in the vicinity of Sewakht mines, district Chitral, Northern Pakistan. Ecotoxicol. Environ. Safe..

[30] Bisson, M. *et al.* Nickel et ses dérivés. *Fiche de données toxicologiques et environnementales des substances chimiques-INERIS*. **296**, (2006).

[31] Singh J, Kalamdhad AS (2011). Effects of heavy metals on soil, plants, human health and aquatic life. Int. J. Res. Chem. Environ..

[32] Ahmad W, Alharthy RD, Zubair M, Ahmed M, Hameed A, Rafique S (2021). Toxic and heavy metals contamination assessment in soil and water to evaluate human health risk. Sci. Rep..

[33] Priya A, Muruganandam M, Ali SS, Kornaros M (2023). Clean-up of heavy metals from contaminated soil by phytoremediation: A multidisciplinary and eco-friendly approach. Toxics..

[34] Wang M, Li S, Chen S, Meng N, Li X, Zheng H (2019). Manipulation of the rhizosphere bacterial community by biofertilizers is associated with mitigation of cadmium phytotoxicity. Sci. Total Environ..

[35] Shi J, Zhao D, Ren F, Huang L (2023). Spatiotemporal variation of soil heavy metals in China: The pollution status and risk assessment. ScI. Total Environ..

[36] Binner H, Sullivan T, Jansen M, McNamara M (2023). Metals in urban soils of Europe: A systematic review. ScI. Total Environ..

[37] Al-Dahar, R., Rabee, A., & Mohammed, R. Calculation of Soil pollution indices with elements in residential areas of Baghdad City. *Revis Bionatura*. **8** (1), 43 (2023). s Note: Bionatura stays neutral with regard to jurisdictional claims in … 2002.

[38] Esmaeilzadeh M, Jaafari J, Mohammadi AA, Panahandeh M, Javid A, Javan S (2018). Investigation of the extent of contamination of heavy metals in agricultural soil using statistical analyses and contamination indices. Hum. Ecol. Risk Assess..

[39] Luo Y, Jia Q (2021). Pollution and risk assessment of heavy metals in the sediments and soils around Tiegelongnan Copper Deposit, Northern Tibet China. J. Chem..

[40] Taghavi M, Darvishiyan M, Momeni M, Eslami H, Fallahzadeh RA, Zarei A (2023). Ecological risk assessment of trace elements (TEs) pollution and human health risk exposure in agricultural soils used for saffron cultivation. Sci. Rep..

[41] Vineethkumar V, Sayooj V, Shimod K, Prakash V (2020). Estimation of pollution indices and hazard evaluation from trace elements concentration in coastal sediments of Kerala, Southwest Coast of India. Bull. Natl. Res. Cent..

[42] Li R, Yuan Y, Li C, Sun W, Yang M, Wang X (2020). Environmental health and ecological risk assessment of soil heavy metal pollution in the coastal cities of Estuarine Bay—A case study of Hangzhou Bay, China. Toxics.

[43] Kijowska-Strugała M, Baran A, Szara-Bąk M, Wiejaczka Ł, Prokop P (2022). Soil quality under different agricultural land uses as evaluated by chemical, geochemical and ecological indicators in mountains with high rainfall (Darjeeling Himalayas, India). J. Soils Sedim..

[44] Tomlinson D, Wilson J, Harris C, Jeffrey D (1980). Problems in the assessment of heavy-metal levels in estuaries and the formation of a pollution index. Helgoländer meeresuntersuchungen..

[45] Muller G (1969). Index of geoaccumulation in sediments of the Rhine River. Geojournal.

[46] Howladar MF, Hossain M, Anju KA, Das D (2021). Ecological and health risk assessment of trace metals in water collected from Haripur gas blowout area of Bangladesh. Sci. Rep..

[47] Mazhar M, Ahmed S, Husain A, Uddin R (2022). Monitoring of trihalomethanes and its cancer risk assessment in drinking water of Delhi City, India. Pollution.

[48] Muhammad N, Nafees M (2018). Geo-chemical investigation and health risk assessment of potential toxic elements in industrial wastewater irrigated soil: A geo-statistical approach. J. Biodiv. Environ. Sci..

[49] Kolo MT, Khandaker MU, Amin YM, Abdullah WHB, Bradley DA, Alzimami KS (2018). Assessment of health risk due to the exposure of heavy metals in soil around mega coal-fired cement factory in Nigeria. Res. Phys..

[50] Adimalla N (2020). Heavy metals contamination in urban surface soils of Medak province, India, and its risk assessment and spatial distribution. Environ. Geochem. Health..

[51] Adamczyk-Szabela D, Wolf WM (2022). The impact of soil pH on heavy metals uptake and photosynthesis efficiency in *Melissa*
*officinalis*, *Taraxacum*
*officinalis,*
*Ocimum*
*basilicum*. Molecules.

[52] Tian K, Huang B, Xing Z, Hu W (2017). Geochemical baseline establishment and ecological risk evaluation of heavy metals in greenhouse soils from Dongtai, China. Ecol. Indicat..

[53] Ennaji W, Barakat A, El Baghdadi M, Rais J (2020). Heavy metal contamination in agricultural soil and ecological risk assessment in the northeast area of Tadla plain, Morocco. J. Sedim. Environ..

[54] Ghasemzadeh GM, Karimi A, Zeinadini A, Khorassani R (2018). Relationship of soil properties with yield and morphological parameters of pistachio in geomorphic surfaces of Bajestan Playa, Northeastern Iran. J. Agric. Sci. Technol..

[55] Eskandari Torghaban M S.A.H. Comparison of surface and subsurface irrigation on the traend of soil sanilinity and growth of pistachio trees in a decade. *J. Protect. Use Water*. **1**, 14–25 (2022).

[56] Fang A, Dong J, An Y (2019). Distribution characteristics and pollution assessment of soil heavy metals under different land-use types in Xuzhou City, China. Sustainability.

[57] Senocak MS, Vehid S (2018). To determine skewness, mean and deviation with a new approach on continuous data. Int. J..

[58] Ahmadi DS, Karami M, Afyuni M (2019). Heavy metal pollution assessment in agricultural soils of Kermanshah province, Iran. Environ. Earth Sci..

[59] Parvez MS, Nawshin S, Sultana S, Hossain MS, Rashid Khan MH, Habib MA (2023). Evaluation of heavy metal contamination in soil samples around Rampal, Bangladesh. ACS Omega.

[60] Ogundele D, Adio A, Oludele O (2015). Heavy metal concentrations in plants and soil along heavy traffic roads in North Central Nigeria. J. Environ. Anal. Toxicol..

[61] Mahmood A, Malik RN (2014). Human health risk assessment of heavy metals via consumption of contaminated vegetables collected from different irrigation sources in Lahore, Pakistan. Arab. J. Chem..

[62] Lemessa F, Simane B, Seyoum A, Gebresenbet G (2022). Analysis of the concentration of heavy metals in soil, vegetables and water around the bole Lemi industry park Ethiopia. Heliyon.

[63] Budovich LS (2021). Effects of heavy metals in soil and plants on ecosystems and the economy. Casp. J. Environ. Sci..

[64] Nicholson FA, Smith SR, Alloway B, Carlton-Smith C, Chambers B (2003). An inventory of heavy metals inputs to agricultural soils in England and Wales. Sci. Total Environ..

[65] Wuana R. A. & Okieimen, F. E. Heavy metals in contaminated soils: a review of sources, chemistry, risks and best available strategies for remediation. ISRN Ecology 2011: 1–20. *Korean J. Soil Sci. Fert*. **34**, 33–41 (2011).

[66] Leu C, Singer H, Müller SR, Schwarzenbach RP, Stamm C (2005). Comparison of atrazine losses in three small headwater catchments. J. Environ. Qual..

[67] McBride M (2013). Arsenic and lead uptake by vegetable crops grown on historically contaminated orchard soils. Appl. Environ. Soil Sci..

[68] Fan J, He Z, Ma LQ, Stoffella PJ (2011). Accumulation and availability of copper in citrus grove soils as affected by fungicide application. J. Soils Sedim..

[69] Tariq S, Shafiq M, Chotana G (2016). Distribution of heavy metals in the soils associated with the commonly used pesticides in cotton fields. Scientifica.

[70] Obaid H, Ma L, Nader SE, Hashimi MH, Sharifi S, Kakar H (2023). Heavy metal contamination status of water, agricultural soil, and plant in the Semiarid Region of Kandahar, Afghanistan. ACS Earth Space Chem..

[71] Su C, Wang J, Chen Z, Meng J, Yin G, Zhou Y (2023). Sources and health risks of heavy metals in soils and vegetables from intensive human intervention areas in South China. Sci. Total Environ..

[72] Hu B, Shao S, Fu T, Fu Z, Zhou Y, Li Y (2020). Composite assessment of human health risk from potentially toxic elements through multiple exposure routes: A case study in farmland in an important industrial city in East China. J. Geochem. Explor..

[73] Ghasemzade A, Karimi A, Ziyaee A, Fotovat A (2021). Pollution assessment and source of selected heavy metals in agricultural Soils, southern Sabzevar, Northeastern Iran. J. Soil Manag. Sust. Prod..

[74] Choobkar N, Parsa F (2018). Assessing the level of heavy metals pollution in the agriculture soil of Zahab plain, Kermanshah (Case study: As, Pb and Cd). J. Environ. Sci. Technol..

[75] Rani J, Agarwal T, Chaudhary S (2021). Heavy metals in agricultural soils of National Capital Region, Delhi: Levels and ecological risk. Curr. World Environ..

[76] Mahmud U, Salam MTB, Khan AS, Rahman MM (2021). Ecological risk of heavy metal in agricultural soil and transfer to rice grains. Discov. Mater..

[77] Ismail NFN, Anua SM, Samad NIA, Hamzah NA, Mazlan N (2020). Heavy Metals in soil and vegetables at agricultural areas in Kota Bharu and Bachok districts of Kelantan, Malaysia. Malays. J. Med. Health Sci..

[78] Eriksson, J. *Concentrations of 61 Trace Elements in Sewage Sludge, Farmyard Manure, Mineral Fertiliser, Precipitation and in Oil and Crops: Citeseer* (2001).

[79] Emurotu J, Onianwa P (2017). Bioaccumulation of heavy metals in soil and selected food crops cultivated in Kogi state, North Central Nigeria. Environ. Syst. Res..

[80] Al-Bagawi A, Mansour D, Aljabri S (2021). Contaminations assessment of some trace metals in agricultural soil and irrigation water analysis at Hail region Saudi Arabia. J. Optoelectron Biomed. Mater..

[81] Hu W, Wang H, Dong L, Huang B, Borggaard OK, Hansen HCB (2018). Source identification of heavy metals in peri-urban agricultural soils of southeast China: An integrated approach. Environ. Pollut..

[82] Lu A, Wang J, Qin X, Wang K, Han P, Zhang S (2012). Multivariate and geostatistical analyses of the spatial distribution and origin of heavy metals in the agricultural soils in Shunyi, Beijing, China. Sci. Total Environ..

[83] Liang J, Feng C, Zeng G, Gao X, Zhong M, Li X (2017). Spatial distribution and source identification of heavy metals in surface soils in a typical coal mine city, Lianyuan, China. Environ. Pollut..

[84] Hu Z, Li J, Wang H, Ye Z, Wang X, Li Y (2019). Soil contamination with heavy metals and its impact on food security in China. J. Geosci. Environ. Protect..

[85] Srivastava V, Sarkar A, Singh S, Singh P, De Araujo AS, Singh RP (2017). Agroecological responses of heavy metal pollution with special emphasis on soil health and plant performances. Front. Environ. Sci..

